# Editorial: Women in plant science - linking genome to phenome

**DOI:** 10.3389/fpls.2024.1454686

**Published:** 2024-09-12

**Authors:** Villő Bernád, Jennifer L. Clarke, Sónia Negrão

**Affiliations:** ^1^ School of Biology and Environmental Science, University College Dublin, Dublin, Ireland; ^2^ International Plant Phenotyping Network, Institute for Bio- and Geosciences, Plant Sciences (IBG-2), Forschungszentrum Jülich GmbH, Jülich, Germany; ^3^ Department of Food Science and Technology, University of Nebraska-Lincoln, Lincoln, NE, United States

**Keywords:** genomics, equity, women, phenomics, plant science

## Introduction

The escalating impacts of climate change are intensifying the urgency for plant scientists to identify and breed plant varieties that are able to withstand increasingly harsh environmental conditions while supporting a growing human population. A key area of focus is in assessing plant performance and linking it with genomic data to speed up breeding efforts and bridge the gap between the lab and field. In recent years, the development of new technologies have led to a rapid drop in the cost of genotyping paired with increased speed (Pootakham, 2023; Thomas et al., 2023; Scheben et al., 2018). The development of high-throughput crop phenotyping technologies has lagged, creating the so called phenotyping bottleneck (Ninomiya, 2022). Traditional phenotyping is slow, destructive, and susceptible to human errors (Gill et al., 2022). The field of plant phenomics aims to overcome these limitations by utilising imaging technologies and high performance computing to make phenotyping faster, cheaper, and more accurate (Kumar and Kaushik, 2023). This special edition underscores research endeavours aiming to achieve these advancements through various means, including the construction of platforms for image capture, the development of segmentation tools for precise data extraction, and the creation of data analysis and management tools to optimize data utilization and accessibility. Moreover, it emphasizes the integration of these tools with genotypic analysis to synergize both fields and extract biologically significant information for plant science and breeding.

Additionally, this Research Topic aims to highlight the invaluable contributions of women in the field. The 2021 UNESCO science report highlights the continued underrepresentation of women in the sciences, particularly in computer science and computational biology (Lewis et al., 2021; Bonham and Stefan, 2017). Deep-rooted biases and gender stereotypes persistently discourage girls and women from pursuing careers in science, technology, engineering, and mathematics (STEM) fields. Therefore, this Research Topic seeks to prominently showcase the outstanding research conducted by female researchers in these domains, shedding light on their significant contributions and advocating for greater gender inclusivity in science.

## Innovative imaging set-ups and platforms

Plant roots play a crucial role in plant adaptations to stress, yet phenotyping them in a high-throughput and non-destructive manner remains challenging (Ye et al., 2023). Claussen et al. introduced “Chamber 8,” a novel high-throughput, non-destructive root phenotyping system. It uses an automated imaging platform to automatically X-ray individual plants in a field-like substance. To overcome possible downstream issues with data processing and analysis, the system takes a holistic approach incorporating data collection, image processing and trait derivation, with complete automatization from initial irrigation to trait computation.

Similarly, measuring maize stem diameter is a critical phenotyping trait essential for yield prediction and resistance assessment (Zhou et al., 2023; Zhang et al., 2020). Manual measurements are laborious, prompting the introduction of a non-invasive, rapid, field-based system. Zhou et al. developed a new method relying on RGB-D cameras that is cost-effective, computationally efficient, and comprehensive, encompassing data collection, processing, and analysis.

## Optimizing data utilization

Image segmentation is often challenging, requiring extensive training and preprocessing, especially with plants due to their varying colours and shapes that change over time. Despite these difficulties, segmentation is essential for accurately reflecting plant conditions through imaging data. Quiñones et al. introduces a new end-to-end unsupervised deep learning framework called Object State Change using Coattention Cosegmentation (OSC-CO2). This framework utilizes coattention-based CNNs and cosegmentation-based dense conditional random fields (CRFs). As the first co-segmentation-based algorithm in plant phenotyping, OSC-CO2 is trained on high-throughput imaging data, including infrared, visible, fluorescence, and multiple views, without requiring additional data annotations.

As the volume of data generated annually continues to increase, managing this data effectively becomes more challenging. Vargas-Rojas et al. addressed this issue by developing two open-source tools: AgTC and AgETL, to enhance data collection and management. AgTC generates standardized data collection templates for use with lab computers or mobile devices, while AgETL handles Extract-TransformLoad (ETL) processes, integrating data from various formats into a database. These tools simplify data management and sharing, offering flexibility without requiring programming knowledge.

The importance of (1) careful calibration set design prior to data collection and (2) hyperparameter optimization for robust model development in future studies is emphasized by Ting et al. The authors conducted nitrogen stress experiments with high-throughput phenotyping in rice using hyperspectral imaging. Their study assessed the ability of HSI-derived data to classify subpopulations and treatment groups over time, identify plant traits with the highest potential for prediction, and evaluated the transferability of models developed within one subpopulation or treatment group to predict values in another. Their findings demonstrate the viability of utilizing canopy-level hyperspectral imaging data to estimate leaf-level nitrogen (N) and carbon:nitrogen ratio (C:N) across diverse rice varieties.

## Linking genome to phenome through advanced technologies

Our understanding of the plant root system is often limited by relying on 2D imaging and a few simple traits to characterize a complex 3D structure. Li et al. used a gel-based optical tomography imaging platform to capture 3D images for maize roots, than measured 84 univariate traits to fully characterize the root system. Through genome-wide association studies (GWAS) they determined that different traits captured distinct root system variation as evidenced by non-overlapping quantitative trait loci (QTLs). These studies corroborate the idea that broadening the range of observed plant traits leads to a more comprehensive understanding of the plant phenome, thereby improving our capability to link it to the underlying genetic diversity.

In another study, Agnew et al. utilize a longitudinal GWAS strategy, with the aim of deepening our understanding of how plants adapt to stress over time. This approach facilitated the identification of early QTLs capable of predicting biomass accumulation in sorghum under cold stress conditions. Sorghum accessions were stratified into distinct clusters based on each heritable trait, showcasing diverse growth profiles. The authors found that the top-performing accessions, exhibiting superior growth-related traits across varying temperatures and time frames, offer avenues for further genetic exploration and breeding endeavours aimed at bolstering biomass yield.

## Conclusion

This special edition includes research articles describing critical advancements in plant phenomics that were made possible by the pivotal role of women researchers in this field. By addressing the multifaceted challenges posed by climate change, the featured studies demonstrate the integration of innovative imaging platforms, advanced data management tools, and comprehensive genomic analyses to accelerate breeding efforts and enhance plant resilience. These interdisciplinary approaches, combining engineering, computer science, bioinformatics, and plant biology, push the boundaries of our understanding and capabilities. Recognizing the contributions of women not only celebrates their achievements but also advocates for greater gender inclusivity, fostering diverse and innovative scientific discoveries for sustainable agricultural practices and food security.
